# A reference-based theory of motivation and effort allocation

**DOI:** 10.3758/s13423-022-02135-8

**Published:** 2022-06-29

**Authors:** Francesco Rigoli, Giovanni Pezzulo

**Affiliations:** 1 Department of Psychology, City, University of London, Northampton Square, London, EC1V 0HB UK; 2 Institute of Cognitive Sciences and Technologies, National Research Council of Italy, Via San Martino della Battaglia 44, 00185 Rome, Italy

**Keywords:** Motivation, Effort, Reference-based model, Subjective value

## Abstract

Motivation is key for performance in domains such as work, sport, and learning. Research has established that motivation and the willingness to invest effort generally increase as a function of reward. However, this view struggles to explain some empirical observations—for example, in the domain of sport, athletes sometimes appear to lose motivation when playing against weak opponents—this despite objective rewards being high. This and similar evidence highlight the role of subjective value in motivation and effort allocation. To capture this, here, we advance a novel theory and computational model where motivation and effort allocation arise from reference-based evaluation processes. Our proposal argues that motivation (and the ensuing willingness to exert effort) stems from subjective value, which in turns depends on one’s standards about performance and on the confidence about these standards. In a series of simulations, we show that the model explains puzzling motivational dynamics and associated feelings. Crucially, the model identifies realistic standards (i.e., those matching one’s own actual ability) as those more beneficial for motivation and performance. On this basis, the model establishes a normative solution to the problem of optimal allocation of effort, analogous to the optimal allocation of neural and computational resources as in efficient coding.

**Supplementary Information:**

The online version contains supplementary material available at 10.3758/s13423-022-02135-8.

Motivation is a key psychological dimension that influences our choices and achievements in every daily activity, such as work, sport, learning, and education. In these and other domains, it is not just skill that matters: Highly motivated employees, sportsmen, and learners invest more effort and achieve better results in their respective domains. There is an emerging consensus that the main role of the motivational system is setting up an optimal level of (physical or cognitive) effort investment, by balancing the costs and benefits of effort—that is, by answering the question: “Is it worth investing effort in this activity, and how much?” (Botvinick & Braver, 2015; Kool & Botvinick, 2014, 2018; Pezzulo et al., 2018b; Shenhav et al., 2013; Shenhav et al., 2017). However, what exactly determines motivation levels—and the decision of whether or not to invest (physical or cognitive) effort—remains far from clear.

A large body of research in experimental psychology and neuroscience has established that objective incentives (e.g., monetary rewards or prestigious achievements) are key factors underlying the motivation to exert effort. This research has shown that motivation to invest physical (Summerside et al., 2018; Yoon et al., 2018) and mental effort (Frömer et al., 2020; Shenhav et al., 2021) increases when the expected reward is greater. Yet, in real-life situations, objective incentives do not often appear sufficient to explain motivational dynamics. This is particularly apparent in fields where measuring effort and performance is simpler, such as skill learning and sport competitions (but could generalize across other domains). For example, it is difficult to remain motivated for the prolonged periods of time necessary to achieve mastery in a skill or to win a championship, despite the incentives being higher near the end of training (Ericsson et al., 1993). Similarly, athletes sometimes lose motivation when they are close to victory (despite the apparent prospect of earning a high reward), or when they are competing with poor opponents—but they could suddenly regain motivation when they are under pressure of losing unexpectedly (Jones & Hardy, 1990). Altogether, this and similar evidence highlights the role of subjective value (SV) in motivational dynamics and effort allocation.

To capture this role, here we advance a novel theory of *reference-based motivation*. In a nutshell, the theory proposes that the value of effort investment corresponds to the difference in SV between the outcome a person expects to achieve with effort minus the outcome expected without effort. As an example, consider an athlete participating in a race expecting that the first place can be achieved by investing effort, whereas the third place is expected to be obtained even without effort. In this example, the value of investing effort will correspond to the difference in SV between the first and the third place, with effort being exerted only if this value surpasses effort costs. Distinguishing our proposal from previous accounts (Botvinick & Braver, 2015; Kool & Botvinick, 2014, 2018; Pezzulo et al., 2018b; Shenhav et al., 2013; Shenhav et al., 2017), we propose that SV is *reference based* (Bhui et al., 2021; Kőszegi & Rabin, 2006; Louie et al., 2013; Rigoli, 2019; Rigoli et al., 2016; Stewart et al., 2006; Woodford, 2012), as it depends on one’s own performance standard (corresponding to prior expectations about one’s accomplishments). As a first approximation, this reflects the performance history: Considering sport as an example, the standard of a novice who has never reached the “top ten” will be significantly lower than the standard of an elite athlete who has won all recent competitions. Furthermore, the theory assumes that it is not just the performance standard, but also the uncertainty about the standard, that determines motivation levels. As we shall see below, by emphasizing the importance of reference-dependent processes (subjective standards and their uncertainty), the theory emphasizes the role of SV in motivational dynamics and effort allocation. In the remainder of the article, we formalize the theory in a computational model and elucidate its functioning.

## The reference-based motivation model

The reference-based motivation model (RBM) aims at offering a general framework to explain how the brain trades off effort in exchange of reward (or for avoiding punishment). For illustrative purposes, the paper examines the RBM in the context of scenarios such as races, championships, or tournaments. However, as emphasized further in the discussion, the model aims at offering a general account that can be applied to any scenario involving trade-offs between effort and incentives.

Let us consider a race or championship where, at any time point *t* (e.g., at any match in a championship), an agent (e.g., a team or player) occupies a specific position *P*_*t*_. The model proposes that, at each time point, the agent makes predictions about which positions will be occupied at the end of the game by exerting different levels of effort. This is indicated by ${P}_T^{\prime }(E)$, reflecting the position at the final time point T predicted by exerting effort level E, the latter being described by a number between 0 and 1 (note that E captures the total effort exerted over a period of time extending from the present time t to the final time point T). For example, an agent might currently occupy the eighth position (*P*_*t*_ = 8) and predict that the 10th, 8th, 5th, and 3rd position will be occupied by exerting 0, 0.1, 0.2, and 0.3 units of effort, respectively (implying that ${P}_T^{\prime }(0)=10,{P}_T^{\prime }(0.1)=8,{P}_T^{\prime }(0.2)=5,{P}_T^{\prime }(0.3)=3\left)\right)$. The model is agnostic about how these predictions arise, although some simple rules will be proposed below.

Moreover, the model proposes that each position P is associated with an SV calculated based on a logistic function and equal to (Rigoli, 2019; Woodford, 2012):

1 $$V(P)=\frac{1}{1+{e}^{\frac{P-\mu }{\sigma }}}$$

This implies that 0 < V(P) < 1 (note that V is higher for better positions). The parameter μ reflects the *standard* about the position occupied at the end. This will normally depend on past experience: For example, an agent accustomed to arriving second in previous games will have μ = 2. The notion of standard is based on previous proposals arguing that outcomes are compared against expectations (Kőszegi & Rabin, 2006). Note that this is different from classical formulations of prospect theory where the standard reflects the status quo (Kahneman & Tversky, 1979; note that more recent work in prospect theory does not necessarily interpret the reference point as status quo; Kőszegi & Rabin, 2006). The parameter σ reflects the uncertainty about the standard. Based on which final position is expected by exerting different effort levels, and based on the ensuing cost, the model proposes that an agent establishes the optimal effort level to be exerted (*E*_*OPT*_):

2 $${E}_{OPT}=\underset{j}{\mathrm{argmax}}\left(V\left({P}_T^{\prime}\left({E}_j\right)\right)-{E}_j\right)$$

In other words, the optimal effort level corresponds to the best option in terms of a trade-off between effort cost and the SV expected to be achieved with that effort level (associated with the final time point T).

Note that, to the aim of highlighting its specificity, the model we have just described relies on various simplifications. First, it assumes that one single time point (the final time point T) matters. Second, it assumes that a single outcome, and not a set of probabilistic outcomes, is predicted for each effort level. Third, it proposes that an agent does not parcel out the future in sequential effort choices, but that it represents the effort assessed during the current choice as encompassing the whole future temporal horizon. Although, as we shall see below, these assumptions facilitate clarity of exposition, it is important to stress that they are appropriate only for a restricted set of conditions. More complex scenarios (not analyzed here) require relaxing these assumptions by considering a transition function that links each combination of effort level and position at time t to a set of probabilistic positions at time t + 1. This transition function can then be integrated with the value function (whereby, according to Eq. 1, each position is imbued with subjective value) and examined by standard methods such as dynamic programming to identify the optimal effort level at time t (Bellman, 1956). We do not pursue this more complex and more general formulation here; this is because focusing on a simpler formulation allows us to better highlight the specific contribution offered by the RBM.

Below, we will illustrate how different parameterizations of the model, such as different standards (captured by the parameter μ) and uncertainties about the standard (captured by the parameter σ), determine motivation and appropriate effort levels in different conditions.

## Model predictions

We illustrate the functioning of the model by focusing on four aspects, concerning the role of the standard parameter μ (Subsection 1 and 2), the role of the uncertainty parameter σ (Subsection 3), the implications of having realistic or unrealistic beliefs about performance (Subsection 4), and the role of learning (Subsection 5).

### How the standard affects motivation and effort

First, we examined the effort level (here, among two effort levels only: effort versus no effort) estimated by each of four car racers who, during a race, occupy various positions (ranging from the 20th to the first) at various time points. Regarding outcome predictions, here all racers predict that two positions will be gained by investing effort and two positions will be lost without effort (e.g., when occupying the 10th position, the outcomes predicted with and without effort will be the 12th and eighth position, respectively). However, the agents have different standards, captured by setting the parameter μ to 15, 10, 5 and 1 for each agent, respectively. We will refer to these agents as to *Agent*_*μ* = 15_, *Agent*_*μ* = 10_, *Agent*_*μ* = 5_, and *Agent*_*μ* = 1_, respectively. Finally, all agents have the same uncertainty about standards (i.e., σ = 2).

**Fig. 1:**
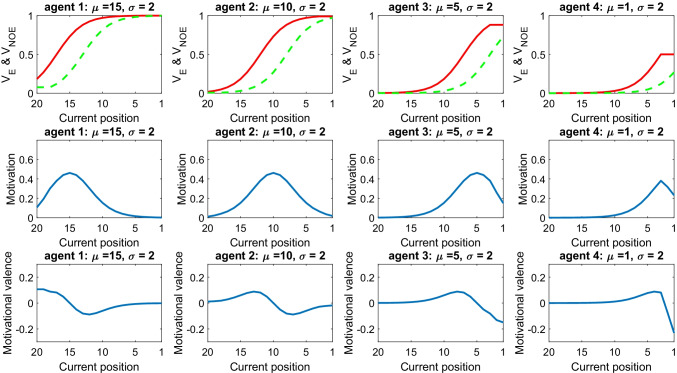
Role of the standard parameter μ. This shows the motivational dynamics of four agents with different standard (15, 10, 5, 1, respectively) but equal uncertainty parameter (i.e., σ = 2). Each column describes a different agent and includes three panels. The top panel shows the subjective value (SV) associated with the outcome expected by exerting effort (red solid line) and expected without effort (green dashed line). The middle panel shows the motivation. The bottom panel shows the motivational valence. Data are shown for different positions (on the *x*-axis). (Colour figure online)

The scenario is described in Fig. 1. The top panels of Fig. 1 illustrate the SV of the positions that agents expect to occupy by exerting effort (*V*_*E*_;red solid lines) and by not exerting effort (*V*_*NOE*_;green dashed lines), both estimated based on Eq. 1. In the model, the greater the difference between the two SVs, the higher the motivation (and the willingness to exert effort) (Rigoli, 2021):

3 $$motivation={V}_E-{V}_{NOE}.$$

For scenarios where more than two effort levels are available, motivation can be defined as follows:

4 $$motivation={V}_{EMAX}-{V}_{NOE},$$

where *V*_*EMAX*_ corresponds to the SV associated with maximal level of effort available, calculated based on Eq. 1.

The motivation estimated in this scenario is illustrated by the middle panels of Fig. 1. Regarding how such motivation translates into the decision of whether or not to exert effort, we simply assume that exerting effort is chosen when the motivation is higher than a threshold (e.g., > .15).

The figure shows that the motivation curves for the four agents peak around their respective standards; for example, it is higher around position 15 for *Agent*_*μ* = 15_ and around position 10 for *Agent*_*μ* = 10_. This is because the reference-based aspect introduces a nonlinearity in motivational computations, rendering the model more sensitive to differences in SV in proximity to the standard (see Fig. 1, top panels).

This scenario predicts that motivation follows an inverted U-shape, namely that it is low far from the standard and high close to the standard. For example, *Agent*_*μ* = 10_ is less motivated in positions much lower than the standard (ranging from 20 to 13) and much higher than the standard (positions 7 to 1), while being highly motivated in proximity of the standard (positions 12 to 8). This prediction is novel and requires being assessed empirically.

The model implies substantial differences in motivation when comparing an elite athlete used to occupy the 5th position (*Agent*_*μ* = 5_) and a novice accustomed to the 15th place (*Agent*_*μ* = 15_). The elite athlete appears very sensitive to differences in position between (for example) the fifth and eighth place, thus being highly motivated when around these places. However, the same athlete assigns almost the same SV to the thirteenth and fourteenth place, those losing motivation when around these positions. The converse is true for the novice.

The scenario reveals that the best athlete (*Agent*_*μ* = 1_) will be most motivated when occupying the third position and not the first. This is banally due to a “boundary effect”: once the first position has been reached, it is not possible to improve it further; therefore, motivation is predicted to be higher in the third position, when further improvement is still possible. The prediction that, for athletes accustomed to victory, the motivation is stronger in proximity of the first place rather than when occupying the first place itself, is novel and requires being tested empirically. Note however that, in real life scenarios, an athlete may set goals which are even higher than just arriving first, for example aiming at beating the world record. Boundary effects would be erased in this example, with the athlete showing strong motivation despite occupying the first place.

The bottom panels of Fig. 1 show that, during a race, an athlete can experience positive or negative motivational valence, defined as:

5 $$\mathrm{Motivational}\ \mathrm{valence}=\left({V}_E-{V}_{CUR}\right)-\left({V}_{CUR}-{V}_{NOE}\right)={V}_E+{V}_{NOE}-2{V}_{CUR},$$

where *V*_*CUR*_ corresponds to the SV associated with the currently occupied position, calculated based on Eq. 1. For scenarios where more than two effort levels are available, motivational valence can be defined as follows:

6 $$\mathrm{Motivational}\ \mathrm{valence}=\left({V}_{EMAX}-{V}_{CUR}\right)-\left({V}_{CUR}-{V}_{NOE}\right)={V}_{EMAX}+{V}_{NOE}-2{V}_{CUR},$$

where *V*_*EMAX*_ corresponds to the SV associated with maximal level of effort available, calculated based on Eq. 1. The motivational valence estimates whether the gain of SV afforded with effort is greater than the loss of SV experienced without effort. A positive motivational valence (values above zero) implies that an athlete is particularly motivated by the prospect of gaining positions, while a negative motivational valence (values below zero) implies that the motivation is more driven by the fear of losing positions. The plot shows that motivational valence is zero when the standard position is occupied (e.g., position 10 for *Agent*_*μ* = 10_), positive for a few positions below the standard (e.g., peaking at position 13 for *Agent*_*μ* = 10_), and negative for a few positions above the standard (e.g., with a minimum at position 7 for *Agent*_*μ* = 10_).

### How contextual factors affect motivation and effort

In the scenario above, we made three simplifying assumptions. First, we assumed that both elite athletes and novices could gain (or lose) the same number of positions by investing (or not investing) effort. However, in most realistic scenarios, better athletes can potentially gain more positions than novices by investing effort (and lose less by not investing effort). Second, we assumed that athletes could gain or lose the same number of positions throughout the race. However, in many sports, athletes can realistically expect to gain more positions at the bottom of the rank (as moving from the fourteenth to the thirteenth position in a race is far easier than moving from the second to the first place) and lose more positions at the top. Finally, we assumed that variations in position are equally likely throughout the race. However, in many sports, athletes can change positions more easily at the beginning of the race (when more time is available) than near the end (when time is running out).

**Fig. 2:**
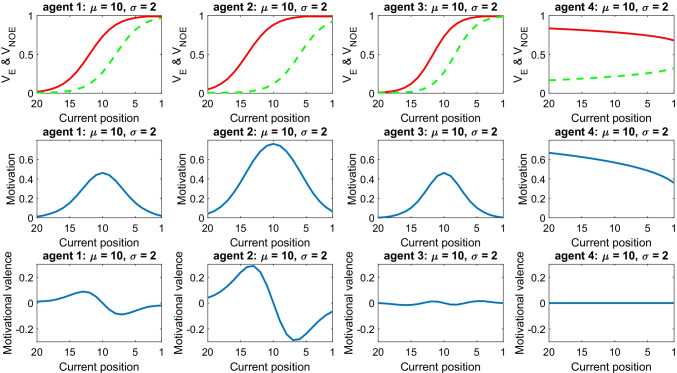
Role of contextual factors. Each column describes an agent under different contextual situations (see main text for explanation). All agents have standard μ = 10 and uncertainty parameter σ = 2. The top panel shows the subjective value (SV) associated with the outcome expected by exerting effort (red solid line) and expected without effort (green dashed line). The middle panel shows the motivation. The bottom panel shows the motivational valence. Data are shown for different positions (on the *x*-axis). (Colour figure online)

Figure 2 illustrates the effects of considering these contextual factors. We simulated four athletes having the same standard (μ = 10) and uncertainty about standard (σ = 2) but dwelling in different situations. The agent simulated first in Fig. 2 (the one on the far left) corresponds to *Agent*_*μ* = 10_ examined in Fig. 1. While this agent can gain or lose two positions with or without effort, respectively, the second agent of Fig. 2 can gain or lose four positions with or without effort, respectively. Here, the differences in SV between the outcomes predicted with or without effort are greater, implying higher motivation. The third agent of Fig. 2 follows slightly more sophisticated rules, as the positions expected to be gained or lost with or without effort are not fixed but depend on the current position. Specifically, for this agent the position predicted by exerting effort is equal to:

7 $${P}_T^{\prime }(E)={P}_t-2-\left({P}_t-\mu \right)/5$$

while the position predicted without effort is:

8 $${P}_T^{\prime }(NOE)={P}_t+2-\left({P}_t-\mu \right)/5$$

In this way, when the athlete occupies the 10th position (the standard), the same number of positions can be gained or lost. However, when the current position is better than the standard (i.e., between the ninth and the first positions) the athlete can lose more positions than those that can be gained. Conversely, when the current position is worse than the standard (i.e., between the 11th and the 20th positions), the athlete can gain more positions than those that can be lost. In this case, the motivation curve increases and decreases more sharply. Finally, while agents from one to three change positions throughout the race, the fourth agent of Fig. 2 always occupies the same position (position 10). In addition, now the outcome predicted by exerting effort becomes closer to the outcome predicted without effort as the competition approaches the end (simply because there is less time to change one’s rank). The result of this is that, for this agent, motivation decreases over time.

This scenario shows that contextual factors (e.g., skill level, self-efficacy and game dynamics) can influence motivation in various ways. For example, an athlete who believes to be able to earn more (less) positions will be on average more (less) motivated, everything else being equal. As explained by prominent theories of motivation in social and sport psychology, a “self-efficacy” belief is fundamental for performance (Bandura, 1977; Moritz et al., 2000); for example, motivation will be poor when an athlete expects effort to be uninfluential. Furthermore, time is also critical: motivation diminishes as time runs out, but it might also rebound when an athlete is informed that some extra time is allowed. Of course, these and other contextual factors are not mutually exclusive, but can be at play concomitantly, thus shaping the complex motivational dynamics observed in sport and other contexts.

### How the uncertainty about standards affects motivation and effort

**Fig. 3:**
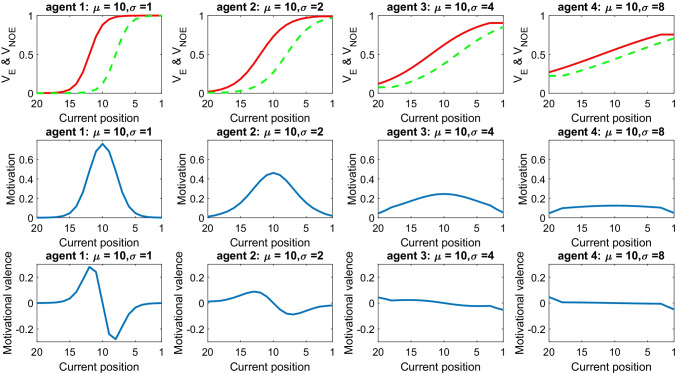
Role of the uncertainty parameter σ. This shows the motivational dynamics of four agents with equal standard (μ = 10) but different uncertainty parameter (1, 2, 4, 8, respectively). Each column describes a different agent and includes three panels. The top panel shows the subjective value (SV) associated with the outcome expected by exerting effort (red solid line) and expected without effort (green dashed line). The middle panel shows the motivation. The bottom panel shows the motivational valence. Data are shown for different positions (on the *x*-axis). (Colour figure online)

In the previous scenarios, the uncertainty parameter was fixed (σ = 2) for all agents. Here, by varying the uncertainty parameter, we illustrate the implications of having different degrees of uncertainty about one’s own standard. Figure 3 describes a scenario where four agents have the same standard (μ = 10) but various levels of uncertainty: σ = 1, 2, 4 and 8 in the four columns, from left to right. The figure shows that the curve of motivation flattens when the uncertainty parameter increases.

From a psychological perspective, the parameter σ captures the level of uncertainty about one’s own performance. This impacts dramatically on motivation in as much as higher uncertainty leads to distributing motivation more evenly across different positions; in other words, to being somewhat motivated in many circumstances, but never highly motivated. Conversely, low uncertainty boosts motivation in proximity of the standard position (in this example, the 10th), while inhibiting motivation elsewhere.

In principle, the uncertainty parameter can be estimated from the history of previous performance. Athletes who find themselves occupying the same position most of the time, will tend to develop low uncertainty, thus being particularly motivated around the typical position and poorly motivated elsewhere. Conversely, athletes used to occupy a wider range of positions will exhibit higher uncertainty, leading to a more widespread motivation across positions, with no extremes. This prediction that variability in past performance shapes the uncertainty parameter and in turn leads to the motivational pattern described in fig. 3 is novel and requires being assessed empirically. Although previous performance is likely to be critical in shaping parameters, other factors might be influential too, such as general psychological traits (e.g., derived from genetic factors or from experience in other contexts).

Finally, while here we focus on the uncertainty about the standard, the model could be extended also to consider the uncertainty about outcome predictions (i.e., about how many places are gained or lost by investing or not investing effort). In this extended model, a higher (lower) uncertainty about outcome predictions would determine lower (higher) motivation, similar to what happens with uncertainty about standards. Furthermore, under the assumption that predicting performance is easier when a race is about to end, the extended model would predict a “burst” of motivation near the end of the race—all other factors being equal.

### How one’s own standards affect performance

This scenario explores which parameter sets are most beneficial in terms of promoting effort. Consider an athlete occupying different positions over time, with the 10th position being the average (and with three positions as *SD*) and each time predicting that a certain number of positions will be gained or lost with effort or without effort, respectively (note that, for the sake of simplicity, here the focus is specifically on scenarios where effort is binarized and where the number of positions that can be gained with effort is equal to the number of positions that can be lost without effort). Here we simulated the athlete’s choice in terms of exerting effort or not (effort was chosen when the motivation exceeded a threshold of 0.15). By simulating different standard (μ) and uncertainty (σ) parameters, we assessed which parameter set promotes maximal effort, thus potentially maximizing performance.

**Fig. 4:**
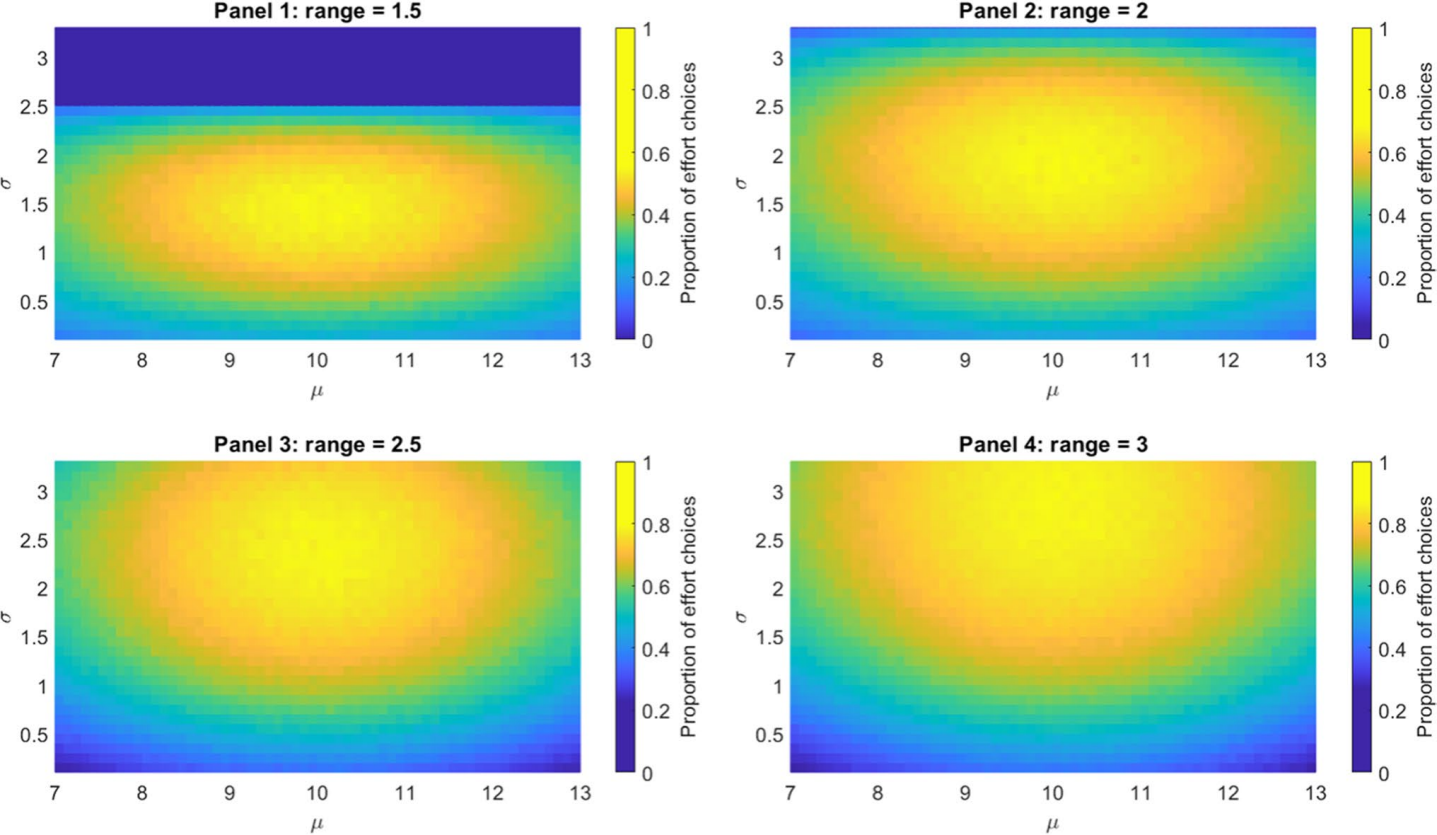
Relationship between parameters and performance. This simulation shows the proportion of effort choices across trials during a race. For each trial, the agent’s position is randomly selected from a normal distribution having the 10th position as mean and three positions as *SD*. Four conditions are simulated, each shown on a different panel (10,000 trials are simulated for each panel) and characterized by a different range (i.e., the difference between the position expected with effort and the position expected without effort): 1.5, 2, 2.5 or 3, respectively. An effort choice occurs when motivation exceeds a threshold of 0.15. (Colour figure online)

Figure 4 describes the proportion of effort choices across time for different parameter sets. Different panels show this when varying the distance between the outcome predicted with effort and the outcome predicted without effort, with this distance being referred to as *range*. The range values examined are 1.5, 2, 2.5 and 3; for example, with a range of 2, the athlete predicts to gain one position with effort and lose one position without exerting effort. Results of this simulation show that, independent of the range, a realistic standard parameter (in this example, μ = 10) leads to maximal effort. Regarding the uncertainty parameter σ, results indicate that maximal effort ensues when the parameter corresponds to the value of the range, namely to 1.5, 2, 2.5, and 3 in panels 1, 2, 3, and 4, respectively. Intuitively, this occurs because, when σ is higher than the range, the outcome predicted by exerting effort and the outcome predicted without effort will tend to be close in SV, thus diminishing the proportion of effort choices. This is most evident in panel 1, where effort is never exerted when σ > 2.5. Conversely, when σ is lower than the range, the outcome predicted by exerting effort and the outcome predicted without effort will be either very close to one another (resulting in no effort choices) or very far (resulting in effort choices). Thus, the overall proportion of effort choices will tend towards 0.5, which is not as high.

This simulation shows that maintaining accurate beliefs about one’s own standard permits to maximize effort investment, hence potentially maximizing performance. At the same time, this simulation indicates that unrealistic standards impair motivation and thus performance. For example, consider an overconfident novice who could realistically achieve the 10th position, but instead expects to win the race (i.e., μ = 1). When occupying the 10th position, as in most cases, the novice will be poorly motivated, thus avoiding effort and exhibiting poor performance (a high standard might be beneficial for the novice when close to the first position; however, this prospect will rarely occur). Excessively low standards impair performance, too: an underconfident athlete with excessively low standards (e.g., μ = 10) despite being able to achieve the third place, will be poorly motivated when occupying the third place, thus preventing the full potential to be realized. The prediction that realistic standards benefit performance is novel and requires being assessed empirically.

The notion that maintaining accurate beliefs about one’s own standard affords an optimal solution to effort investment can be assessed within a normative perspective. Investing effort has benefits in terms or positions gained, but it has also costs; thus, effort can be interpreted as a limited resource to be invested only when useful. The RBM implies that, if one is correctly tuned to the “natural statistics” of performance, effort should be invested to a larger degree in proximity of the average position. This is because, in natural settings, it is around the average position where effort can earn the most (this occurs under the assumption that the outcomes obtained with or without effort differ the most around the average position). This argument links to the notion of *efficient coding*, maintaining that the brain faces the problem of allocating limited computational resources for encoding sensory stimuli. A solution to this problem consists in adapting sensory systems to the statistical properties of their signals and hence allocating more resources to process stimuli that are typical in an environment (Barlow, 1961; Simoncelli & Olshausen, 2001; Wei & Stocker, 2015). Accordingly, previous research has established that sensory neurons in visual (Laughlin, 1981), auditory (Smith & Lewicki, 2006) and olfactory (Kostal et al., 2008) systems best encode those signals that occur most frequently.

Our proposal suggests that the optimal allocation of effort might parallel the optimal allocation of computational resources as in efficient coding, and hence ensure that effort-investment decisions adapt to environmental statistics. This proposal links well with other recent research advancements in the fields of behavioural economics and neuroeconomics. A strong parallel can be drawn with the notion of *efficacy of control* in the expected value of control model (Shenhav et al., 2021). The rationale is that control is a limited (and costly) resource and hence one should invest it when it matters the most. For an athlete that most often occupies the 10th position, this average position is where investing control is most efficacious in making a difference—and hence where more effort should be invested. Another manifestation of the capability of the brain to adapt to environmental statistics is *adaptive gain control*, or the adaptation (e.g., normalization) of the gain of neural firing of sensory systems to background levels of stimulation (Carandini & Heeger, 2012). Previous research has shown that sensory gain can adapt on a fast time scale during decision-making, coming to reflect the average of the available evidence (Cheadle et al., 2014). Furthermore, sensory gain can be modulated to improve sensory information processing when both high choice accuracy and fast responses are desirable—but this comes at a cost, akin to the costs of investing effort or control. Hence, it is optimal to invest more effort in enhancing sensory gain when there is more reward to earn (Manohar et al., 2015).

Taken together, these diverse lines of research suggest that similar optimality principles might underlie the allocation of limited and costly resources like effort, control, and sensory gain—and that all the brain systems that regulate cost-based computations benefit from a good fit to environmental statistics, akin to what emerges from our simulation in Fig. 4.

### How standards are shaped by experience

**Fig. 5:**
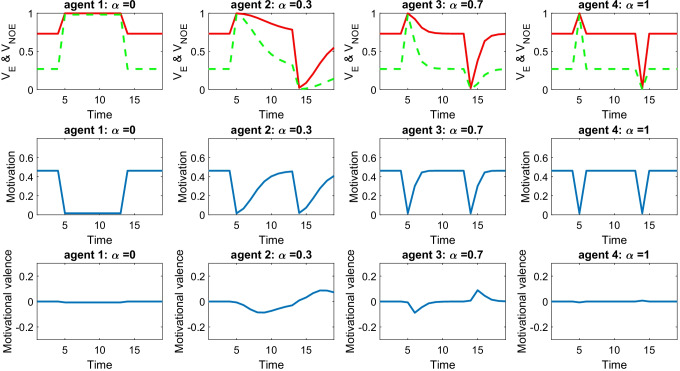
How the standard is learnt from experience. This shows the motivational dynamics of four agents with different learning rate (0, 0.3, 0,7, 1, respectively). All agents occupy the 15th position during the initial four time points, reaching the fifth position from Time Point 5 to 13, and moving back to the 15th position from Time 14 to 20 (agents start with the same standard μ_1_ = 15). Each column describes a different agent and includes three panels. The top panel shows the subjective value (SV) associated with the outcome expected by exerting effort (red solid line) and expected without effort (green dashed line). The middle panel shows the motivation. The bottom panel shows the motivational valence. Data are shown for different time points (on the *x*-axis). (Colour figure online)

Where do the parameters of the RBM come from? Experience arguably plays a key role. Here we explore how experience might shape the standard parameter μ. A possibility is that standards can be updated even within a single race, implying that the very same position can be assessed very differently at the start or at the end of a race. Let assume that *μ*_*t*_ corresponds to the standard parameter for time t. The following delta-rule can be proposed to calculate *μ*_*t* + 1_, corresponding to the standard parameter for time t + 1:

9 $${\mu}_{t+1}={\mu}_t+\alpha \left({P}_t-{\mu}_t\right),$$

where the parameter α is a learning rate bounded between zero and one (remember that P_t_ indicates the position occupied at time t). Let us examine the impact of having different values for the learning rate parameter. Figure 5 describes four agents all occupying the 15th position during the initial four time points, reaching the fifth position from Time Point 5 to 13, and moving back to the 15th position from time 14 to 20. Agents start with the same standard *μ*_1_ = 15 but have different learning rates, ranging from zero to one (from the left to the right of the figure). Figure 5 shows that agents completely lose motivation when jumping to the fifth position (see middle panels), as now all outcomes look great independent of whether effort is exerted or not. However, after some trials, all agents except the first one (the agent with null learning rate) adjust their standards and acquire motivation again. How many trials are needed for this to occur depends on the learning rate: when this is equal to one (as for agent four), one single trial is enough. A similar effect occurs later in the sequence when agents jump back to the 15th position. Now, all outcomes look grim independent of whether effort is exerted or not, and motivation is lost. Yet again, all agents except the first one (the agent with null learning rate) adjust their standards and reacquire motivation.

This scenario captures phenomena in sports and other contexts where events might change dramatically someone’s perspective over time. Our example indicates that a novice starting with low standards (μ = 15) who unexpectedly occupies the fifth place might update standards accordingly (with μ = 5 now). The novice might end up being disappointed by a final placement (say, the sixth place) that would have appeared as remarkable at the start. This can be referred to as the “Cinderella effect”: At the beginning of the tale, Cinderella is not excessively concerned about not joining the ball. Later, she is allowed to join, but shortly afterwards she is denied again this possibility. Now, the prospect of not joining the ball comes with extreme disappointment, although this prospect was not even imagined at the beginning of the tale. These examples stress how experience influences one’s standards and the ensuing motivation, and how this influence might act in a remarkably short time frame (e.g., even within the same race).

## Discussion

Excellence in domains such as sport, business, and teaching, requires not just skill but also high motivation, and thus high willingness to exert effort. What determines motivation, and how this can be enhanced, is therefore a key question in all the above, and many other, domains. We advance a novel theory and computational model of motivation and effort allocation, arguing that a reference-based SV is attributed to predictions of what can be achieved by exerting different levels of effort. Within this framework, the standard about performance and the uncertainty about this standard emerge as key in determining how valuable effort is in different circumstances. In keeping with the ancient Greek aphorism γνῶϑι σεαυτόν (“know thyself”), the theory predicts that setting up realistic standards, and holding them with appropriate confidence, is conducive of optimal motivation and effort allocation.

Previous research has established that motivation is influenced by incentives in such a way that the willingness to exert effort is greater when effort can achieve higher reward (Botvinick & Braver, 2015; Kool & Botvinick, 2014, 2018; Pezzulo et al., 2018b; Shenhav et al., 2013; Shenhav et al., 2017). Our theory highlights subjective factors that influence motivational dynamics and effort investment. It proposes that motivation arises from estimating the SV that can be afforded with effort minus the SV expected without effort. Crucially, SV is constructed in a reference-based manner, thus rendering the value of external incentives fundamentally dependent on subjective factors. In this way, the theory explains scenarios where extrinsic factors appear as insufficient for explaining effort investment, such as conditions when motivation is lost or gained although the objective rewards at stake remain the same. By the same token, our proposal links well to other theories that highlight the importance of intrinsic factors, such as self-efficacy (Bandura, 1977; Moritz et al., 2000), self-determination, and the desire to demonstrate competence (Nicholls, 1984), in determining motivation—and it provides a formal ground to express these intrinsic factors.

Furthermore, the theory helps making sense of the subjective feelings associated with different outcomes. Considering again the example of sport, the proposal is that the SV associated with a given position in a race (e.g., the third) depends on one’s own standard, implying that reaching this position will be evaluated very differently by different individuals (e.g., athletes expecting the tenth and first position, respectively). The same holds for supporters of a sportsman or team who unexpectedly exceeds or fails to meet their expectations. The theory fits with common reports of athletes who consistently maintain high standards—namely, reports that for these athletes the negative feelings associated with (unexpected) failures are much greater than the positive feelings associated with (expected) successes. As the famous tennis player Andre Agassi wrote: “A win doesn’t feel as good as a loss feels bad, and the good feeling doesn’t last long as the bad. Not even close” (Agassi, 2011). To avoid such negative feelings, a strategy is maintaining very low standards, and some athletes may be tempted to adopt this strategy even at the expense of performance. According to the theory, a similar deterioration of performance arises when standards are too high. Altogether, realistic standards—namely, those that match the actual athlete’s ability, are identified by the theory as those more beneficial for motivation and performance.

Although we have so far assumed that the higher the motivation and the better the performance, this assumption might need to be nuanced. This is because the theory distinguishes between a positive and negative motivational valence, the former at play when one is driven by gaining positions, the latter when one is driven by avoiding losing positions. A possibility is that, when the motivational valence is negative, stress or Pavlovian aversive reactions such as fight/flight or freezing might arise, with a detrimental impact upon performance (Jones & Hardy, 1990). This possibility is supported by empirical evidence showing that, in some aversive circumstances, people often “choke under pressure” (Baumeister, 1984) and display poor performance despite being highly motivated.

A key assumption of the RBM is that SV depends on a logistic function (Rigoli, 2019; Woodford, 2012). This formulation has several advantages over alternatives such as standard expected utility (e.g., based on an exponential value function) or prospect theory (Kahneman & Tversky, 1979). Expected utility theory is unsuitable to capture reference effects, which are the focus of the RBM; thus, an exponential value function (or analogous functions proposed by standard expected utility) appears as being inappropriate in this context. On the contrary, by proposing that outcomes are compared against the status quo, prospect theory offers an influential framework for describing reference effects (Kahneman & Tversky, 1979). However, at least three shortcomings of prospect theory are worth emphasizing. First, the concept of status quo appears as inappropriate to explain why the current position influences motivation: If the status quo was the critical factor, then motivation would not vary as a function of the current position (because the current position can be arguably regarded as the status quo). On the contrary, treating the reference point as an expectation (as captured by the standard parameter of the logistic function) allows the RBM to predict that motivation varies as a function of the current position (Kőszegi & Rabin, 2006). Second, the value function proposed by prospect theory does not formally fit with the notion of efficient coding, while a logistic function does (Rigoli, 2019; Woodford, 2012). This is important because, by relying on a logistic function, the RBM proposes a principled way to interpret effort allocation as analogous to efficient coding. Third, in prospect theory the reference point alone is responsible for reference effects; there is no parameter analogous to the uncertainty σ. This precludes prospect theory to capture reference effects observed empirically (Rigoli, 2019; Rigoli et al., 2016) ensuing from the variability of a distribution, and not only from its average (and examined here when assessing the role of the uncertainty parameter σ). For all these reasons, we argue that a logistic function, more than the function proposed by prospect theory, offers valuable insight on reference effects at play during motivational dynamics.

Prospect theory offers a compelling explanation of the empirical phenomenon of loss aversion (Kahneman & Tversky, 1979). A classical experiment in this domain asks participants to choose between a safe outcome of zero and a 50–50 gamble associated with either winning a monetary amount or losing the same amount (Tom et al., 2007). Here, participants manifest a preference for the safe option. In prospect theory, this phenomenon is captured by an asymmetry in the value function when comparing gains versus losses. Is the RBM at all compatible with loss aversion? Although an exhaustive answer to this question goes beyond the scope of the present paper, we argue that the RBM is not necessarily incompatible with loss aversion, for the following reason. A common assumption (shared by prospect theory) is that, for participants, the reference point corresponds to an outcome of zero. However, this assumption might turn out to be misleading, and participants’ reference point might actually correspond to something different. Assuming, instead, that participants usually have a reference point smaller than a zero outcome (captured, in the RBM, by a standard parameter smaller than zero), the RBM produces loss aversion (e.g., a preference for a safe outcome of zero over a 50–50 gamble associated with either winning a monetary amount or losing the same amount). Based on this consideration, an interesting research avenue is to fully explore the RBM in the domain of loss aversion.

The RBM might offer insight about impaired motivational processes as observed in mental illness (Rigoli & Martinelli, 2021; Rigoli et al., 2021). These impairments can be interpreted as arising from alterations in the model parameters. For example, an excessively high uncertainty about standards may produce a general loss of motivation, thus failing to incentivize (cognitively or physically) effortful behaviour—as evident for example in apathy and Parkinson’s disease (McGuigan et al., 2019). In the brain, an increased uncertainty parameter might be the result of dysfunctions affecting neuromodulators such as dopamine, as often observed in these disorders (Salamone & Correa, 2012).

To give an idea of how the RBM can be applied to real-life conditions, the paper has focused on scenarios such as races or championships. However, the model aims at offering a general explanation of how motivation and the willingness to exert effort arise. Formally, it is straightforward to extend the model to any context where effort needs to be traded off with other incentives (rewards or punishments): Simply, in the equations above *position* P needs to be replaced with *incentive* I, where the latter is now a real number (with positive and negative numbers corresponding to reward and punishment, respectively). Everything else remains the same. This more general formulation can be applied to a variety of problems in the literature such as foraging or work performance.

Finally, we highlight limitations of the RBM in its current version. First, the RBM assumes that one single time point (the final time point T) matters, while several real-life scenarios require integrating outcomes at multiple time points. Second, the current version of the RBM assumes that, for each effort level, a single outcome is predicted; more realistically, the brain might consider a set of outcomes, each associated with a specific probability. Third, this model is agnostic about the neural processes that underly motivation and effort allocation. Evidence indicates that an important role in these processes is played by neuromodulators such as dopamine, serotonin, and noradrenaline (Basten et al., 2010; Meyniel et al., 2013; Niv et al., 2007; Shenhav et al., 2021; Silvetti et al., 2018; Skvortsova et al., 2014). Fourth, the RBM assumes that the brain explicitly predicts the outcomes associated with different effort levels. In the context of a race, this requires complex representations such as about one’s own ability and the ability of other players; in fact, the brain might not rely on such complex representations, but rather on simpler heuristics. Many effort allocation settings are social (cooperative or competitive) and require predicting the performance of other people (e.g., of competitors) and not just of oneself. Previous research has established that, during social tasks, humans infer other people’s intentions and, on this basis, predict other people’s actions (Knoblich & Flach, 2001; Pezzulo et al., 2018a; Tomasello et al., 2005; Yoshida et al., 2008). The question of how precisely the brain constructs representations of other people’s motives and abilities remains outside the scope of the RBM. For example, the brain might simply rely on “group-level” models, without examining each single agent individually (Khalvati et al., 2019; Pezzulo et al., 2013).

In summary, we introduce a theory and computational model where motivation and effort allocation arise from reference-dependent evaluation processes. The theory sheds light on apparently puzzling phenomena in sports and other contexts where, unexpectedly, the will to exert effort raises or declines despite no apparent change in objective incentives. The theory argues that these phenomena arise because of entertaining specific standards about performance and specific levels of confidence about these standards. The RBM could be potentially applied to a variety of more specific scenarios, for example for predicting motivation, emotions, and performance of athletes and players—and more generally, to study motivational dynamics in a broad variety of contexts such as sports, work, learning, and education.

## Supplementary Information

ESM 1 (M 2 kb)

ESM 2 (M 2 kb)

ESM 3 (M 2 kb)

ESM 4 (M 1 kb)

ESM 5 (M 2 kb)

## Data Availability

N/A

## Code Availability

The code is available as supplementary material.

## References

[CR1] Agassi, A. (2011). *Open*. J’ai Lu.

[CR2] Bandura A (1977). Self-efficacy: Toward a unifying theory of behavioral change. Psychological Review.

[CR3] Barlow, H. B. (1961). Possible principles underlying the transformation of sensory messages. *Sensory Communication*, 217–234.

[CR4] Basten U, Biele G, Heekeren HR, Fiebach CJ (2010). How the brain integrates costs and benefits during decision making. Proceedings of the National Academy of Sciences of the United States of America.

[CR5] Baumeister RF (1984). Choking under pressure: Self-consciousness and paradoxical effects of incentives on skillful performance. Journal of Personality and Social Psychology.

[CR6] Bhui R, Lai L, Gershman SJ (2021). Resource-rational decision making. Current Opinion in Behavioral Sciences.

[CR7] Botvinick M, Braver T (2015). Motivation and cognitive control: From behavior to neural mechanism. Annual Review of Psychology.

[CR8] Carandini M, Heeger DJ (2012). Normalization as a canonical neural computation. Nature Reviews Neuroscience.

[CR9] Cheadle S, Wyart V, Tsetsos K, Myers N, de Gardelle V, Herce Castañón S, Summerfield C (2014). Adaptive gain control during human perceptual choice. Neuron.

[CR10] Ericsson KA, Krampe RT, Tesch-Römer C (1993). The role of deliberate practice in the acquisition of expert performance. Psychological Review.

[CR11] Frömer R, Lin H, Wolf CKD, Inzlicht M, Shenhav A (2020). When effort matters: Expectations of reward and efficacy guide cognitive control allocation. BioRxiv.

[CR12] Jones J, Hardy LE (1990). Stress and performance in sport.

[CR13] Kahneman D, Tversky A (1979). Prospect theory: An analysis of decision under risk. Econometrica.

[CR14] Khalvati K, Park SA, Mirbagheri S, Philippe R, Sestito M, Dreher J-C, Rao RPN (2019). Modeling other minds: Bayesian inference explains human choices in group decision-making. Science Advances.

[CR15] Knoblich G, Flach R (2001). Predicting the effects of actions: Interactions of perception and action. Psychological Science.

[CR16] Kool W, Botvinick M (2014). A labor/leisure tradeoff in cognitive control. Journal of Experimental Psychology. General.

[CR17] Kool W, Botvinick M (2018). Mental labour. *Nature human*. Behaviour.

[CR18] Kostal L, Lansky P, Rospars J-P (2008). Efficient olfactory coding in the pheromone receptor neuron of a moth. PLoS Computational Biology.

[CR19] Kőszegi B, Rabin M (2006). A model of reference-dependent preferences. The Quarterly Journal of Economics.

[CR20] Laughlin S (1981). A simple coding procedure enhances a neuron’s information capacity. Zeitschrift Für Naturforschung c.

[CR21] Louie K, Khaw MW, Glimcher PW (2013). Normalization is a general neural mechanism for context-dependent decision making. Proceedings of the National Academy of Sciences.

[CR22] Manohar SG, Chong TT-J, Apps MAJ, Batla A, Stamelou M, Jarman PR, Bhatia KP, Husain M (2015). Reward pays the cost of noise reduction in motor and cognitive control. Current Biology.

[CR23] McGuigan S, Zhou S-H, Brosnan MB, Thyagarajan D, Bellgrove MA, Chong TT-J (2019). Dopamine restores cognitive motivation in Parkinson’s disease. Brain.

[CR24] Meyniel F, Sergent C, Rigoux L, Daunizeau J, Pessiglione M (2013). Neurocomputational account of how the human brain decides when to have a break. Proceedings of the National Academy of Sciences of the United States of America.

[CR25] Moritz SE, Feltz DL, Fahrbach KR, Mack DE (2000). The relation of self-efficacy measures to sport performance: A meta-analytic review. Research Quarterly for Exercise and Sport.

[CR26] Nicholls JG (1984). Achievement motivation: Conceptions of ability, subjective experience, task choice, and performance. Psychological Review.

[CR27] Niv Y, Daw ND, Joel D, Dayan P (2007). Tonic dopamine: Opportunity costs and the control of response vigor. Psychopharmacology.

[CR28] Pezzulo, G., Donnarumma, F., Dindo, H., D’Ausilio, A., Konvalinka, I., & Castelfranchi, C. (2018a). The body talks: Sensorimotor communication and its brain and kinematic signatures. *Physics of Life Reviews.*10.1016/j.plrev.2018.06.014 10.1016/j.plrev.2018.06.014 30072239

[CR29] Pezzulo G, Iodice P, Ferraina S, Kessler K (2013). Shared action spaces: A basis function framework for social re-calibration of sensorimotor representations supporting joint action. Frontiers in Human Neuroscience.

[CR30] Pezzulo G, Rigoli F, Friston K (2018). Hierarchical active inference: A theory of motivated control. Trends in Cognitive Sciences.

[CR31] Rigoli F (2019). Reference effects on decision-making elicited by previous rewards. Cognition.

[CR32] Rigoli F (2021). Political motivation: A referent evaluation mathematical model. Journal of Social and Political Psychology.

[CR33] Rigoli F, Martinelli C (2021). A reference-dependent computational model of anorexia nervosa. Cognitive, Affective, & Behavioral Neuroscience.

[CR34] Rigoli F, Martinelli C, Pezzulo G (2021). The half-empty/full glass in mental health: A reference-dependent computational model of evaluation in psychopathology. Clinical Psychological Science.

[CR35] Rigoli F, Friston KJ, Martinelli C, Selaković M, Shergill SS, Dolan RJ (2016). A Bayesian model of context-sensitive value attribution. ELife.

[CR36] Salamone JD, Correa M (2012). The mysterious motivational functions of mesolimbic dopamine. Neuron.

[CR37] Shenhav A, Botvinick MM, Cohen JD (2013). The expected value of control: An integrative theory of anterior cingulate cortex function. Neuron.

[CR38] Shenhav, A., Fahey, M. P., & Grahek, I. (2021). *Decomposing the motivation to exert mental effort*. PsyArXiv. 10.31234/osf.io/yrd8n 10.1177/09637214211009510 PMC8528169 34675454

[CR39] Shenhav A, Musslick S, Lieder F, Kool W, Griffiths TL, Cohen JD, Botvinick MM (2017). Toward a rational and mechanistic account of mental effort. Annual Review of Neuroscience.

[CR40] Silvetti M, Vassena E, Abrahamse E, Verguts T (2018). Dorsal anterior cingulate-brainstem ensemble as a reinforcement meta-learner. PLoS Computational Biology.

[CR41] Simoncelli EP, Olshausen BA (2001). Natural image statistics and neural representation. Annual Review of Neuroscience.

[CR42] Skvortsova V, Palminteri S, Pessiglione M (2014). Learning to minimize efforts versus maximizing rewards: Computational principles and neural correlates. Journal of Neuroscience.

[CR43] Smith EC, Lewicki MS (2006). Efficient auditory coding. Nature.

[CR44] Stewart N, Chater N, Brown GDA (2006). Decision by sampling. Cognitive Psychology.

[CR45] Summerside EM, Shadmehr R, Ahmed AA (2018). Vigor of reaching movements: Reward discounts the cost of effort. Journal of Neurophysiology.

[CR46] Tom SM, Fox CR, Trepel C, Poldrack RA (2007). The neural basis of loss aversion in decision-making under risk. Science.

[CR47] Tomasello M, Carpenter M, Call J, Behne T, Moll H (2005). Understanding and sharing intentions: The origins of cultural cognition. Behavioral and Brain Sciences.

[CR48] Wei X-X, Stocker AA (2015). A Bayesian observer model constrained by efficient coding can explain ‘anti-Bayesian’ percepts. Nature Neuroscience.

[CR49] Woodford M (2012). Prospect theory as efficient perceptual distortion. American Economic Review.

[CR50] Yoon T, Geary RB, Ahmed AA, Shadmehr R (2018). Control of movement vigor and decision making during foraging. Proceedings of the National Academy of Sciences.

[CR51] Yoshida W, Dolan RJ, Friston KJ (2008). Game theory of mind. PLoS Computational Biology.

